# Safety Outcomes of Hybrid Open Chest Transvenous Lead Extraction: A Multicenter Experience

**DOI:** 10.1111/pace.70118

**Published:** 2025-12-22

**Authors:** Nadeev Wijesuriya, Helena Lytton Cobbold, Keisha Kellman, Nikolaos Vogiatzakis, Felicity De Vere, Sandra Howell, Alphonsus Liew, Jonathan M. Behar, Paolo Bosco, Gianluca Lucchese, Vias Markides, Neil R. Grubb, Vishal Mehta, Steven A. Niederer, Tom Wong, Christopher A. Rinaldi

**Affiliations:** ^1^ King's College London London UK; ^2^ Guy's and St Thomas's NHS Foundation Trust London UK; ^3^ Edinburgh Heart Centre Edinburgh UK; ^4^ Imperial College London London UK; ^5^ Alan Turing Institute London UK; ^6^ Cleveland Clinic London UK

**Keywords:** cardiac implantable electronic device, cardiac surgery, extraction

## Abstract

**Background:**

Hybrid open chest transvenous lead extraction (TLE), combining surgical and endovascular techniques, may be utilized in patients requiring concomitant cardiac surgery or with high‐risk features for endovascular extraction. Outcome data in this population remains sparse.

**Aims:**

To evaluate procedural outcomes and identify predictors of complications in patients undergoing elective hybrid open chest TLE.

**Methods:**

A retrospective multicenter cohort study was conducted, including 40 patients between 2017 and 2025 across three UK tertiary centers. Patients undergoing emergency surgical conversion were excluded. Baseline, procedural, and outcome data were collected. Primary outcomes were in‐hospital mortality and complications, graded using a modified Delphi classification. Logistic regression was used to identify predictors of any or severe (Delphi grade ≥3) complications.

**Results:**

The mean age was 61.9 ± 17 years; 62.5% were male. Median lead dwell time was 10.5 years. The indication was infection in 65% of cases. Concomitant valve intervention was performed in 77.5% of cases. Clinical procedural success was 97.5%. In‐hospital mortality was 2.5%, with a rate of severe complications of 15%, and an overall complication rate of 37.5%. Multivariable analysis identified chronic lung disease as an independent predictor of severe complications (OR 102.2, *p* = 0.03). Atrial fibrillation was an independent predictor of any complication (OR 5.83, *p* = 0.04), driven primarily by post‐procedure rhythm intervention. Lead dwell time and EROS classification were not associated with adverse outcomes.

**Conclusion:**

Hybrid open chest TLE demonstrates high procedural success and despite significant morbidity, has acceptable mortality rates. Chronic lung disease independently predicts complications and should guide patient selection and perioperative planning.

**Abbreviations**
CIED: cardiac implantable electronic device
CRT: cardiac resynchronization therapy
ICD: implantable cardioverter defibrillator
PPM: permanent pacemaker
TLE: transvenous lead extraction

## 1 Introduction

Cardiac implantable electronic devices (CIED) have transitioned from a bradycardia therapy in the early days of the field, to a vital pillar of treatment in heart failure and ventricular tachyarrhythmia, with the advent of implantable cardioverter defibrillators (ICD) and cardiac resynchronization therapy (CRT) [1]. With an increase in CIED implantation rates to over 1.2 million annually worldwide [2], the long‐term management of transvenous leads is becoming more vital.

CIEDs become infected or fail at rates of 2%–4% annually [3, 4], and device infection carries a class I indication for lead extraction in the HRS consensus statement [2]. The treatment for lead failure is less clear; however, there is evidence that increased transvenous lead burden can adversely affect outcomes with regard to infection, thrombosis, tricuspid regurgitation, and inappropriate shocks [5, 6, 7, 8]. This evidence has led to the consideration that extraction may be preferable to abandonment in the case of lead malfunction, reflected in the large European Electra Registry, where 47% of extractions were for non‐infective indications [9].

While open heart surgery was originally required in all patients requiring lead extraction, in the last three decades transvenous lead extraction (TLE) via endovascular approaches has usurped this as the preferred treatment option in the majority of cases, with a lower morbidity than median sternotomy, and emergent complication rates of less than 2% in contemporary cohorts [9]. There are, however, selected patients who continue to benefit from an elective “hybrid” procedure, where endovascular extraction is performed in combination with an open‐surgical approach [10]. This is primarily employed when concomitant intervention is required, such as valve replacement or debulking of infective vegetations, but can also be considered in cases where lead dwell time is very prolonged, and the risk of superior vena cava injury is high [11].

The current data for hybrid open chest lead extractions is limited to case series and small cohort studies, which show varying results, with in‐hospital mortality ranging from 0% to 40% [10, 12, 13]. We aimed to add to this evidence base through the largest dedicated study analyzing outcomes in these patients to date.

## 2 Methods

### 2.1 Patient Selection

Research was conducted in accordance with the Declaration of Helsinki, with institutional ethical review board approval. This was a multicenter retrospective cohort analysis of patients from three high‐volume (over 30 cases per year) tertiary extraction centers in the United Kingdom. Patients were identified from existing institutional databases and theatre records. Consecutive patients from 2017 to 2025 were included. Patients met the inclusion criteria if they underwent a procedure using a hybrid approach (that is, endovascular combined with open chest surgical intervention) electively. Patients were not included if emergent sternotomy was performed to treat a complication of a planned fully endovascular extraction. For identified patients, baseline characteristics, procedural data, and outcomes were collected from electronic health care records. The data that support the findings of this study are available from the corresponding author upon reasonable request.

### 2.2 Procedure

Pre‐procedure, all patients were discussed in a multidisciplinary team meeting with input from cardiologists and cardiac surgeons. If the indication was infection, the case was also discussed in the infective endocarditis MDT. All patients underwent pre‐procedure CT chest to plan access. Cases were performed in dedicated hybrid theatres with joint input from cardiologists and cardiac surgeons. General anesthesia, invasive monitoring, and peri‐procedural transesophageal echocardiography were employed.

The procedural workflow involved initially opening the chest, followed by the endovascular stage of the extraction, with cardiopulmonary bypass (CPB) on standby. Subsequently, CPB was commenced for the surgical portion of the procedure.

Following extraction plus concomitant intervention (if any), patients either received permanent CIED or temporary epicardial leads as per standard cardiac surgical protocol if it was determined that a permanent device was not suitable for implantation at this stage. Pericardial and pleural drains were left in situ at the surgeons’ discretion, and negative pressure vacuum dressings were used for the CIED pocket if there was purulent exudate.

Post‐procedure, patients were recovered in cardiac intensive care settings as per the institution's standard operating protocols. Post‐procedure chest radiographs and transthoracic echocardiograms were performed to monitor for immediate complications.

### 2.3 Outcomes

The primary outcomes assessed were in‐hospital complications and in‐hospital mortality. Complications were identified from electronic health records. These were classified as mild, moderate, or severe based on prior published categorization of post‐cardiac surgery complications, which were formulated using a Delphi process. Consensus of the classification of the complications in this cohort were reached by the physicians in our group specializing in extraction procedures (JB, PB, GL, VM, NG, TW, and AR) [14]. Clinical procedural success was defined as per the 2018 EHRA consensus statement, that is, retention of <4 cm of the lead when this part does not increase the risk of undesired outcomes [14]. Follow‐up data extended to 6 months post‐procedure in all patients.

### 2.4 Statistical Analysis

Baseline characteristics are summarized by mean ± standard deviation (SD) for continuous variables with a normal distribution tested by the Shapiro–Wilk test, median ± interquartile range (IQR) for variables with a skewed distribution, and frequency distribution (%) for categorical variables. Comparisons between subgroups were made using Student's *t*‐tests for continuous variables if normally distributed and Wilcoxon rank‐sum tests if not normally distributed, and Pearson's Chi‐squared tests for categorical variables.

Predictors of “any” or “severe” in hospital complications were assessed using logistic regression models. Univariate analysis was performed for baseline variables collected, with variables included in the multivariable analysis if *p* < 0.1. Euroscore 2 [15] was tested as a predictor in a univariate model, but was not included in the multivariate model due to multicollinearity with multiple other variables which form part of the composite score (chronic lung disease, prior sternotomy, and number of valve interventions). The Hosmer–Lemeshow test was used to test the goodness‐of‐fit for the logistic regression model. A subgroup analysis was also performed grouping patients by EROS classification [11] which was used to categorize patients based on multiple factors. In this consensus‐based system, patients are classified into EROS 1 (low risk), EROS 2 (intermediate risk), and EROS 3 (high risk) based on multiple components, including baseline factors such as LV function, presence of congenital heart disease, and infective indication. This system relies heavily on lead dwell time, with patients classified into the highest risk EROS 3 grade if the system has a pacing lead >15 years old or an ICD lead >10 years old.

Analyses were conducted with Stata Statistical Software Package Release 18 (StataCorp LLC, College Station, TX). A *p* value of less than 0.05 was considered significant.

## 3 Results

### 3.1 Baseline Characteristics

Forty patients in total were identified. Baseline characteristics are summarized in Table 1. Male patients comprised 62.5% of the cohort. The mean age was 61.9 ± 17 years. The mean LVEF was 55%±9%. Seventeen (42.5%) patients had received a prior sternotomy. Seventy‐five percent of patients had standard pacemakers, with the remainder having complex devices (ICD or CRT‐D). Seventy percent of patients had two indwelling transvenous leads, 15% had three or more. The median lead dwell time was 10.5 years (5.5–17). The median Euroscore 2 was 5.5% (2.9–8.7).

**TABLE 1:** Baseline characteristics.

**Baseline characteristic**	**Total cohort (*n* = 40)**
Male sex	25 (62.5%)
Age (mean, years)	61.9 ± 17
Body mass index (kg/m^2^)	27.43 ± 6
Ischemic heart disease	2 (5%)
Atrial fibrillation	19 (47.5%)
Anticoagulation	20 (50%)
Diabetes	6 (15%)
Chronic lung disease	6 (15%)
Prior sternotomy	17 (42.5%)
Left ventricular ejection fraction	55% ± 9
Euroscore 2 (median and IQR, %)	5.48 (5.78)
Device Type ‐ Pacemaker ‐ ICD ‐ CRT‐P ‐ CRT‐D	30 (75%) 9 (22.5%) 0 (0%) 1 (2.5%)
Number of leads ‐ 1 ‐ 2 ‐ 3 ‐ 4	6 (15%) 28 (70%) 5 (12.5%) 1 (2.5%)
Lead dwell time (median and IQR, years)	10.5 (12)

### 3.2 Procedure Indications

**FIGURE 1:**
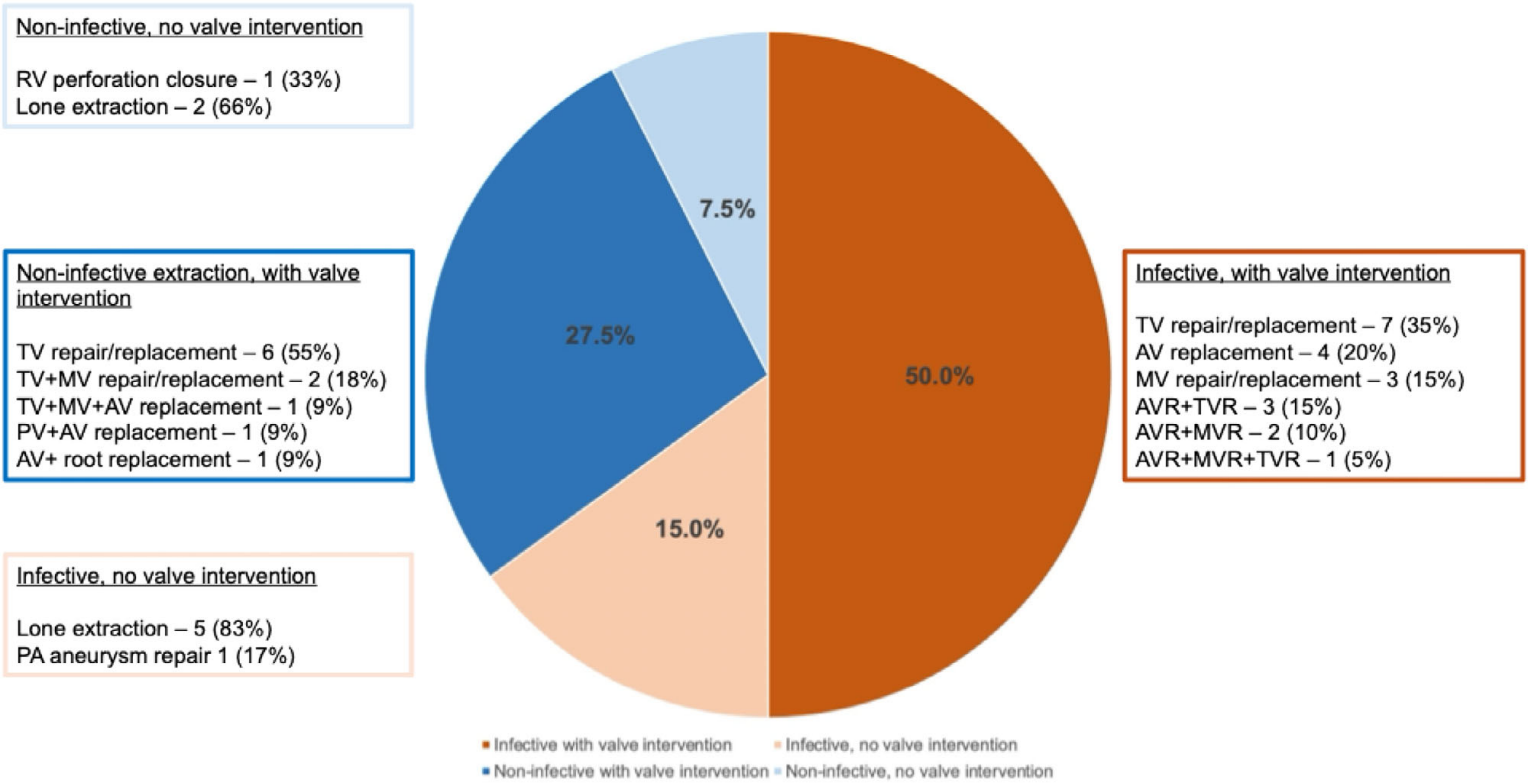
Procedure indications. AV, aortic valve; AVR, aortic valve replacement; MV, mitral valve; MVR, mitral valve replacement; PA, pulmonary artery; PV, pulmonary valve; RV, right ventricle; TV, tricuspid valve; TVR, tricuspid valve replacement. [Colour figure can be viewed at wileyonlinelibrary.com]

Broadly, procedure indication could be classified into four categories: infective extraction plus valve intervention (50%); infective extraction without valve intervention (15%); non‐infective extraction with valve intervention (27.5%); and non‐infective extraction without valve intervention (7.5%). Overall, 65% of patients had an infective indication. The most common concomitant procedure was tricuspid valve intervention, which was undertaken in 50% of the cohort. In all of these cases, tricuspid valve dysfunction was evident at baseline and was not caused by the TLE component of the procedure. Ten patients (25%) underwent multiple valve replacements. Full indications are summarized in Figure 1.

Of the nine patients that underwent lone extraction without valve intervention, the indications for a hybrid approach were as follows: implantation of epicardial pacing systems (*n* = 4), RV perforation repair in a patient on dual anti‐platelets following recent primary PCI (*n* = 1), repair of mycotic PA aneurysm (*n* = 1), congenital heart disease (*n* = 1), prior failed percutaneous attempt (*n* = 1), and long lead dwell time (*n* = 1).

### 3.3 Procedure Details

The rate of clinical procedural success was 97.5% (39 out of 40 patients). With regards to extraction tools, laser sheaths and mechanical sheaths were used in 37.5% and 30% of patients, respectively. Overall, three patients (7.5%) received surgical intervention directly related to lead extraction—one patient required SVC repair using a bovine patch following lead removal, one required RV perforation repair, and one underwent surgical removal of a retained RV lead fragment which snapped during attempted TLE. In terms of re‐implanted devices, 23 patients (57.5%) received permanent epicardial systems, 8 (22.5%) received transvenous systems, 2 (5%) received leadless RV pacemakers, and 2 (5%) received subcutaneous ICDs. The remaining 5 patients (12.5%) did not receive a replacement permanent system, but temporary epicardial leads alone as per standard post‐surgical care. Of the 17 patients who did not receive an epicardial system, 14 (82%) received a concomitant surgical intervention alongside extraction (13 received valve intervention, 1 underwent RV perforation repair).

The median procedure duration was 246 min (160–315). The median duration of hospital stay was 19.5 days (10–39).

### 3.4 Acute Complications

There was one instance of in‐hospital mortality from multiple organ failure (2.5%). Five patients (12.5%) experienced a non‐fatal Delphi grade 3 (severe) complication. The rate of Delphi 2 (moderate) and Delphi 1 (mild) complications was 20% and 2.5% respectively. Twenty‐five patients (62.5%) had no in‐hospital complications.

The full list of complications is summarized in Table 2. Seven patients (17.5%) had more than one complication. One patient (2.5%) required re‐sternotomy for bleeding, and two patients (5%) had acute strokes.

**TABLE 2:** Procedural complications.

**Delphi grade 1 (mild)**		**Delphi grade 2 (moderate)**		**Delphi grade 3/4 (severe/death)**	
Surgical emphysema (conservative management)	1 (2.5%)	Pulmonary embolism	1 (2.5%)	Pericardial effusion secondary to myocardial tear	2 (5%)
		acute kidney injury	2 (5%)	Ventricular fibrillation	1 (2.5%)
		Bleeding requiring transfusion	2 (5%)	RV failure requiring ECMO	1 (2.5%)
		Pulmonary oedema	2 (5%)	Bleeding requiring re‐sternotomy	1 (2.5%)
		Pneumonia	3 (7.5%)	Stroke	2 (5%)
		Pleural effusion requiring drainage	3 (7.5%)	Renal failure requiring dialysis	1 (2.5%)
		Atrial arrhythmia	3 (7.5%)	Death (multiorgan failure)	1 (2.5%)
		Acute lead revision	1 (2.5%)		

Abbreviation: ECMO, extra‐corporeal membrane oxygenation.

### 3.5 Predictors of Acute Complications

Logistic regression analysis was performed to determine predictors of procedural complications. For the outcome of Delphi grade 3+ complications (severe or death), univariate analysis (Table 3) revealed AF, chronic lung disease, prior sternotomy, Euroscore 2, and number of valve interventions (i.e., number of valves repaired or replaced concomitantly during the extraction procedure) as predictors with *p* < 0.1. With the exception of Euroscore 2, which was excluded for multicollinearity, these variables were carried forward to the multivariable model (Table 4). Multivariable analysis revealed chronic lung disease to be the only independent predictor of severe complications in this cohort (Odds Ratio [OR] 102.16, *p* = 0.03). Four out of five patients (80%) with chronic lung disease in this cohort experienced a Delphi 3+ complication (two strokes, RV failure requiring extra‐corporeal membrane oxygenation [ECMO], and multi‐organ failure leading to death).

**TABLE 3:** Univariate logistic regression analysis for severe complications or death.

**Variable**	**Odds ratio**	**Standard error**	***z***	***p* value**	**Confidence interval**
Male sex	3.50	4.02	1.09	0.28	0.37–33.30
Age	1.01	0.03	0.45	0.65	0.96–1.07
BMI	1.02	0.07	0.33	0.74	0.89–1.17
AF	7.14	8.21	1.71	0.09	0.75–68.00
Diabetes	3.75	3.81	1.30	0.19	0.51–27.5
Chronic lung disease	32.00	36.22	3.06	0.002	3.48–294.20
Prior sternotomy	9.16	10.56	1.92	0.06	0.96–87.80
LV ejection fraction	0.96	0.05	−0.71	0.48	0.87–1.07
Number of pacing leads	0.51	0.41	−0.83	0.41	0.11–2.47
Maximum lead dwell time	1.02	0.05	−0.42	0.68	0.92–1.13
Defibrillator lead presence	0.48	0.56	−0.63	0.52	0.05–4.65
System infection	3.09	3.56	0.98	0.33	0.32–29.53
Valve intervention (yes/no)	1.54	1.80	0.37	0.71	0.15–15.17
Number of valve interventions	3.52	0.04	2.04	0.04	1.05–11.81
Presence of vegetation	2.25	2.19	0.83	0.41	0.33–15.25
Vegetation size	1.02	0.04	0.41	0.68	0.93–1.11
Euroscore 2	1.187	0.09	2.25	0.02	1.03–1.38

**TABLE 4:** Multivariable logistic regression analysis for severe complications or death.

**Variable**	**Odds ratio**	**Standard error**	***z***	***p* value**	**Confidence interval**
AF	0.40	0.74	−0.50	0.62	0.01–14.92
Chronic lung disease	102.16	215.99	2.19	0.03	1.62–6441.42
Prior sternotomy	31.18	68.41	1.57	0.12	0.42–2297.42
Number of valve interventions	7.59	9.03	1.71	0.09	0.74–78.15

Regression analysis was also carried out to determine predictors of *any* in‐hospital complication. Univariate analysis (Table 5) revealed AF, chronic lung disease, Euroscore 2, valve intervention (yes versus no), and number of valve interventions to be predictors with *p* < 0.1. Euroscore 2 and valve intervention (yes versus no) were not included in the multivariable model to avoid multicollinearity with the number of valve interventions. As such, AF, chronic lung disease, and the number of valve interventions were included in the multivariable model (Table 6). This revealed AF to be a significant independent predictor of any complication (OR 5.83, *p* = 0.04).

**TABLE 5:** Univariate analysis for any complication.

**Variable**	**Odds ratio**	**Standard error**	***z***	***p* value**	**Confidence interval**
Male sex	2.16	1.53	1.09	0.28	0.53–8.67
Age	1.02	0.02	1.07	0.28	0.98–1.06
BMI	0.93	0.06	−1.19	0.24	0.83–1.04
AF	10.28	8.07	2.97	0.003	2.21–47.84
Diabetes	1.83	1.64	0.68	0.50	0.32–10.52
Chronic lung disease	12.00	13.90	2.15	0.03	1.24–116.17
Prior sternotomy	2.03	1.35	1.07	0.29	0.55–7.47
LV ejection fraction	0.95	0.04	−1.36	0.18	0.87–1.02
Number of pacing leads	0.67	0.37	−0.73	0.47	0.22–2.00
Maximum lead dwell time	0.97	0.04	−0.66	0.51	0.90–1.05
Defibrillator lead presence	0.27	0.24	−1.50	0.14	0.05–1.49
System infection	0.44	0.30	−1.19	0.24	0.11–1.70
Valve intervention (yes/no)	6.60	7.38	1.68	0.09	0.73–59.21
Number of valve interventions	3.29	1.79	2.19	0.03	1.13–9.54
Presence of vegetation	1.01	0.04	0.41	0.68	0.93–1.10
Vegetation size	0.95	0.77	−0.06	0.95	0.19–4.72
Euroscore 2	1.14	0.08	2.02	0.04	1.00–1.30

**TABLE 6:** Multivariable analysis for any complication.

**Variable**	**Odds ratio**	**Standard error**	***z***	***p* value**	**Confidence interval**
AF	5.83	4.98	2.06	0.04	1.09–31.12
Chronic lung disease	7.96	10.14	1.63	1.03	0.65–96.65
Number of valve interventions	2.45	1.56	1.41	0.16	0.71–8.54

There were 19 patients with pre‐existing AF, of whom 13 experienced a complication (68%). In three of these patients, the complication was post‐operative atrial arrhythmia requiring acute rhythm control intervention. To determine if this was the driver of the significant multivariable regression result, a sensitivity analysis was performed with these three patients excluded (Table 7). Regression analysis (*n* = 37) revealed that when these patients were excluded, AF remained a univariate predictor (OR 7.7, *p* = 0.01), however, was no longer a significant independent predictor in multivariable analysis (OR 3.0, *p* = 0.25). Chronic lung disease again was shown to be a significant independent predictor in this sensitivity analysis (OR 13.57, *p* = 0.05).

**TABLE 7:** Sensitivity analysis excluding three patients where the complication was post‐operative atrial arrhythmia. Multivariable logistic regression of any complication.

**Variable**	**Odds ratio**	**Standard error**	***z***	***p* value**	**Confidence interval**
AF	3.00	2.86	1.15	0.25	0.46–19.44
Chronic lung disease	13.57	17.82	1.98	0.05	1.03–178.20
Number of valve interventions	3.08	2.10	1.66	0.10	0.81–11.69

Nine patients had cardiac vegetations. The presence of vegetations and vegetation size were not significant predictors of any or severe complications in this cohort (Tables 3 and 5).

### 3.6 Subgroup Analysis by EROS Grade

**FIGURE 2:**
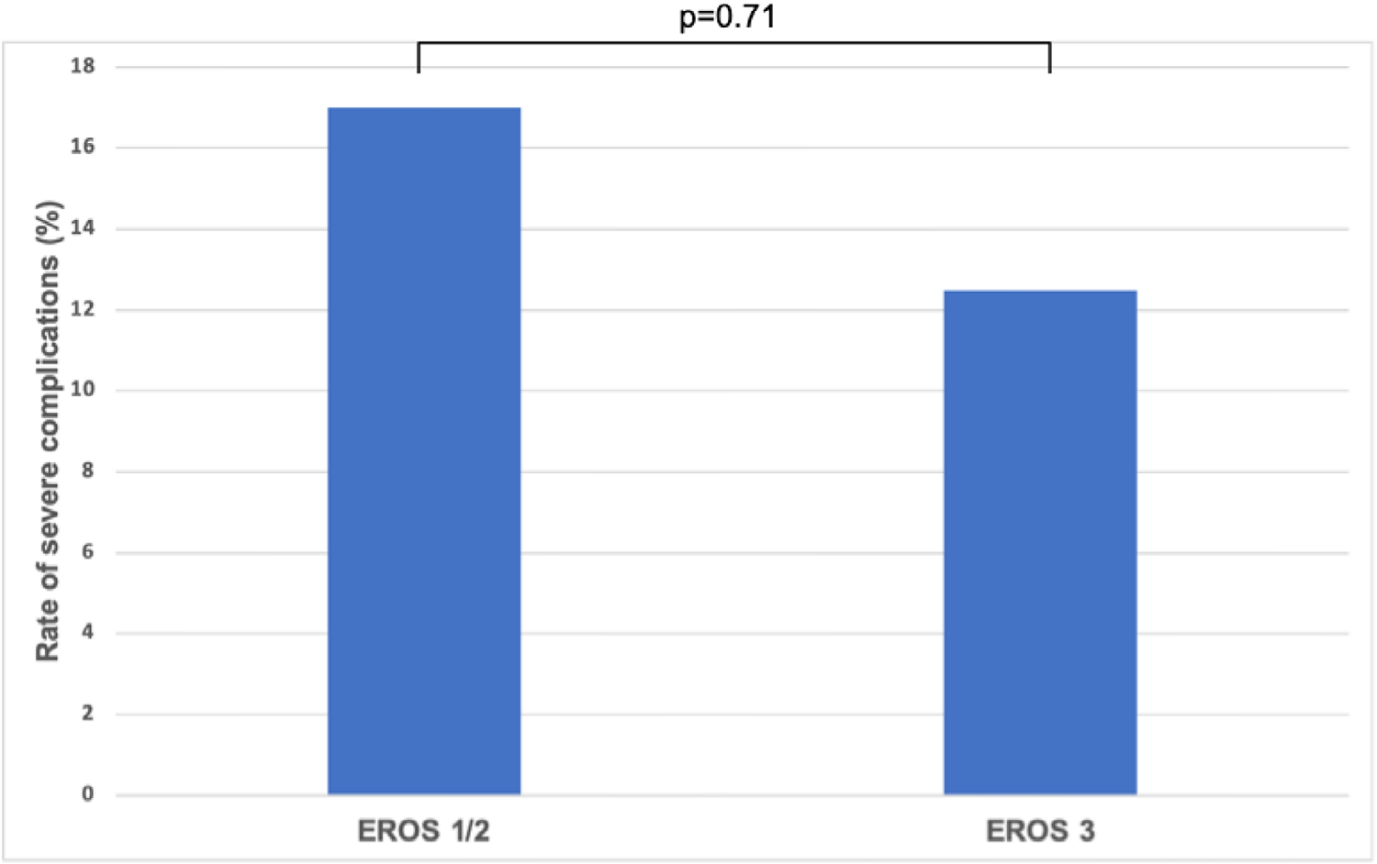
Rate of severe complications in EROS 3 versus EROS 1/2 patients. [Colour figure can be viewed at wileyonlinelibrary.com]

A subgroup analysis by EROS grade was performed. EROS grade 3 (indwelling PPM lead over 15 years or ICD lead >10 years) has previously been shown to be a significant predictor of severe adverse events for endovascular TLE. The analysis showed that in this cohort, there was no significant difference in rates of severe complications between the 16 “EROS 3” patients and the 24 “EROS 1–2 patients” (Figure 2, *p* = 0.71), despite a significant difference in the mean dwell time between the two subgroups (20.3 ± 6.7 years versus 7.08 ± 3.8 years, *p* < 0.01).

### 3.7 Follow‐Up Outcomes

One further patient died during the follow‐up period from non‐cardiac causes, making the overall 6‐month mortality rate 5%. Two patients received re‐intervention during follow‐up—one required a transvenous RV lead revision for late displacement, and one required a new transvenous pacing system following failure of the epicardial system.

## 4 Discussion

We present an analysis of the largest cohort of patients receiving hybrid open chest TLE to date, and the first to evaluate patient characteristics which predict safety outcomes. Our findings were as follows:

1. The clinical procedural success rate was 97.5%.
2. The in‐hospital mortality rate was 2.5%. The rate of severe complications (Delphi 3+) was 15%. The rate of any complication was 37.5%.
3. Chronic lung disease was a significant independent predictor of severe complications. AF was a significant independent predictor of any complication, driven by patients requiring acute post‐operative rhythm control.
4. Lead dwell time and EROS grade did not significantly impact safety outcomes.

### 4.1 Efficacy Outcomes

We report a clinical procedural success rate of 97.5%. This compares favorably to prior studies. In a cohort of 29 patients who received open chest extraction, Gupta et al. report a clinical success rate of 97% [10]. Similarly, the reported clinical success rate in the Electra Registry for transvenous lead extraction was 98.5% [9]. Of note, the median lead dwell time in this cohort was 10.5 years compared to the Electra Registry, where it was 5 years. In Electra, dwell time of greater than 10 years was identified as a predictor of clinical failure in the registry, with an odds ratio of 4.0 (2.2–7.26, *p *< 0.01). While not a direct comparison, our study suggests that open chest extraction may have higher success rates in patients with long lead dwell times, which may reflect that this approach allows for direct dissection of older leads, which could be adherent to the SVC or myocardium. Open extraction may also have added benefit in patients with infection, where direct visualization may aid in the complete removal of insulation or other retained materials which may act as a nidus. Larger studies would be required to assess such efficacy outcomes.

### 4.2 Safety Outcomes

We report an in‐hospital mortality rate of 2.5%. Prior smaller studies of hybrid extraction have reported mortality rates varying from 0% to 40% [12, 16, 17, 18], highlighting the heterogeneity in the current evidence base. The overall procedural major complication and mortality rate in the Electra registry was 1.7% (mortality 0.5%) with all‐cause major complications, including death at 2.7% and all‐cause in‐hospital mortality of 1.4%, mainly related to infection. In Electra, outcomes were primarily driven by very favorable outcomes in the lowest risk EROS 1 patients (0.5%), whereas the mortality rate was 2.7% in EROS grade 2 or 3 patients.

Despite a relatively favorable mortality rate, we report a significant rate of morbidity, with a major complication rate of 15%, and an overall complication rate of 37.5%. This is higher than that observed with endovascular TLE, where major complication rates are generally between 1% and 2% [9]. A retrospective analysis of 1151 patients undergoing TLE from 2000 to 2019 in our center reported a major complication rate of 1.7%, a minor complication rate of 7.3%, and a procedural success rate of 98.9% [19]. The Gupta et al. study [10] of open chest extraction reported a major complication rate of 7% (2 out of 29 patients requiring re‐sternotomy for bleeding).

Certain complications in this cohort (e.g., pleural effusion, surgical emphysema) were directly related to surgical interventions; however, the majority of complications (e.g., stroke, atrial arrhythmia, pneumonia, and acute kidney injury) are not exclusive to surgical procedure or TLE, rather are likely a function of a more prolonged procedure, higher number of interventions and increased recovery times.

As expected, surgical extraction carries a higher morbidity than endovascular TLE, especially with the incremental risks of concomitant procedures. However, this does not appear to translate to a significantly higher mortality rate. It may be that while open surgery exposes patients to increased overall complications, this is to an extent offset by the ability to treat life‐threatening events such as SVC tear or myocardial perforation in a more controlled manner than endovascular procedures, thus balancing the overall mortality profile. This is exemplified by one of the patients in this cohort, where an SVC laceration was repaired in a controlled manner with a bovine patch following laser lead extraction of 15‐year‐old pacing leads.

### 4.3 Predictors of Adverse Events

This is the first study to investigate factors predicting adverse outcomes in open chest extraction. We report that chronic lung disease was the only significant predictor of severe Delphi 3+ complications in this cohort. This is in line with well‐established data from larger studies in cardiac surgery. In a study of 454 patients, Ponomarev et al. reported that chronic obstructive pulmonary disease (COPD) was significantly associated with 1‐year mortality, even after adjustment for Euroscore (Hazard ratio 4.13, *p* < 0.01) [20]. Similarly, in a study of 1169 patients (41% with COPD) receiving cardiac surgery, operative mortality was 2% in those without COPD, compared to 6.7% in those with COPD [21]. In the largest study to date, severe COPD was significantly associated with early mortality in a cohort of over 13,000 patients undergoing isolated coronary artery bypass surgery (adjusted OR, 2.31 *p* = 0.01) [22].

While this evidence seems compelling, it should be noted that there may be several unmeasured factors associated with lung disease that may act as confounders, for example, RV function, nutritional status, and frailty. As such, care should be taken when risk‐stratifying individual patients based solely on this diagnosis.

While not statistically significant, there was a trend towards increased complication rates with multiple valve interventions, which is in line with previous evidence, due to the increased complexity and procedure times [23]. Similarly, AF is also known to be a risk factor for post‐operative adverse events [24, 25, 26, 27], and in this cohort, the primary driver of this was rhythm‐related interventions.

Notably, in this study, we did not observe the same trends seen in endovascular TLE cohorts, where lead dwell time is a major factor impacting outcomes. Lead dwell time greater than 10 years was a significant predictor of major complications in the Electra Registry (OR 3.54, *p* < 0.01) [9] and subgroup analysis of this registry showed that EROS 3 grade patients (defined as lead dwell time >15 years for pacing leads and >10 years for defibrillator leads) had significantly increased risk of complications compared to EROS 1 patients [11]. As theorized above, this is likely because an open chest approach, while increasing morbidity, is protective against life‐threatening events such as SVC tear or tamponade, with the ability to repair lesions in a controlled manner, and with the routine placement of pericardial and pleural drains to reduce the consequences of post‐procedure effusions.

## 5 Clinical Perspective

This study adds to a sparse body of evidence related to hybrid open chest extraction procedures. Although endovascular TLE has largely supplanted open surgery as first‐line treatment, we give evidence here that these procedures can be performed with mortality rates comparable to endovascular TLE in higher‐risk patients. When undertaken in the correct setting, with appropriate expertise, dedicated hybrid theatres and cardiac intensive care facilities, hybrid extraction remains suitable for selected patients, particularly those requiring concomitant valve intervention. It also provides the opportunity to place permanent epicardial pacing systems in those where transvenous systems are contraindicated, for example, patients with endocarditis.

Our study also shows that in these patients, conventional risk factors for cardiac surgery such as chronic lung disease are likely to have a greater bearing on risk than factors associated with endovascular extraction risk such as lead dwell time. In patients with severe chronic lung disease, it may be that treatments such as the AngioVac system (Vortex Medical, US) for percutaneous debulking of infective tricuspid vegetations may be reasonable alternatives to sternotomy [28].

Future progress in this field may be in the form of minimally invasive surgical techniques. Novel approaches such as mini‐sternotomy, subxiphoid, and mini‐thoracotomy have been reported in a single‐center case series of extractions, with varying complication rates for each technique [13]. Advancements and more widespread use of minimally invasive procedures may be a path to improving operative morbidity of open extractions, while continuing to reap the benefits of having direct access to the leads.

## 6 Limitations

As with any retrospective observational design, there are inherent biases in our study. In particular, selection bias is likely to be impacting these results to a degree, as the presence of infection was not shown to be significantly associated with complications, a factor which was predictive in the Electra Registry. It may be that higher‐risk endocarditis patients were not offered open surgery due to operative risk—as such care must be taken when translating this evidence to clinical practice. In addition, the small sample size reduces the power of the study and increases the fragility index, particularly in view of the relatively small number of severe complications. This is an issue with all research in this field, where the evidence consists of small cohorts, mostly single‐center, with a highly heterogeneous patient population. To solidify this evidence base, the most appropriate study design would likely be a large multicenter registry, replicating Electra, in order to more reliably quantify efficacy, safety, and determine the predictors of adverse outcomes with increased power. On this note, on the basis of our findings, more detailed information on quantifiable lung function would be beneficial in future work when studying this association prospectively, as this data was not available for this study. In addition, future work would benefit from a direct comparison with a reference TLE patient database from the institutions during the same time span, which unfortunately was not available for this study.

## 7 Conclusion

Hybrid open chest lead extraction, while associated with significant morbidity, can be performed effectively for indicated patients in the appropriate setting to identify and treat complications expeditiously. Lung disease appears to be a significant risk factor for post‐operative complications, and should be considered when making individual treatment plans.

## Funding

No specific funding was received for this manuscript. N.W., F.D.V., and S.H. are supported by the Wellcome/EPSRC Centre for Medical Engineering (WT203148/Z/16/Z). N.W. has received funding from the British Heart Foundation (BHF) (FS/CRTF/22/24362) and travel funding from EBR Systems. N.G. receives consultation fees from Philips. SAN acknowledges support from the UK Engineering and Physical Sciences Research Council (EP/M012492/1, NS/A000049/1, EP/P01268X/1), the BHF (PG/15/91/31812, PG/13/37/30280, SP/18/6/33805), US National Institutes of Health (NIH R01‐HL152256), and European Research Council (ERC PREDICT‐HF 864055). C.A.R. receives research funding and/or consultation fees from Abbott, Medtronic, Boston Scientific, Philips, and EBR Systems.

## Conflicts of Interest

The authors declare no conflicts of interest.
